# Recycled PET Nanofibers for Water Filtration Applications

**DOI:** 10.3390/ma9040247

**Published:** 2016-03-30

**Authors:** Nicole E. Zander, Margaret Gillan, Daniel Sweetser

**Affiliations:** United States Army Research Laboratory, Weapons and Materials Research Directorate, Aberdeen Proving Ground, Aberdeen, MD 21005, USA; margaret.gillan2.ctr@mail.mil (M.G.); sweetser@udel.edu (D.S.)

**Keywords:** electrospinning, nanofibers, recycled PET, water filtration, antimicrobial

## Abstract

Water shortage is an immediate and serious threat to our world population. Inexpensive and scalable methods to clean freshwater and wastewater are in high demand. Nanofiber filtration membranes represent a next generation nonwoven filter media due to their unique properties. Polyethlyene terephthalate (PET) is often used in the packaging of water and other commonly used materials, leading to a large amount of plastic waste often with limited incentive for recycling (few value-added uses). Here, we present work in the generation of nanofiber liquid filtration membranes from PET plastic bottles and demonstrate their use in microfiltration. PET nanofiber membranes were formed via solution electrospinning with fiber diameters as low as *ca.* 100 nm. Filtration efficiency was tested with latex beads with sizes ranging from 30 to 2000 nm. Greater than 99% of the beads as small as 500 nm were removed using gravity filtration. To reduce biofouling, the mats were functionalized with quaternary ammonium and biguanide biocides. The biguanide functionalized mats achieved 6 log reduction for both gram negative and gram positive bacteria.

## 1. Introduction

In spite of the widespread availability of plastic recycling centers, only approximately 5% of the 32 million tons of plastic in the United States are recycled [1,2]. One of the major contributors to the large volume of plastics in the waste stream is water bottles. Polyethlyene terephthalate (PET) is the most common polyester thermoplastic and is widely used in fibers for clothing, containers for food and liquid packaging, and thermofoaming for manufacturing [3]. Recently, there has been research in the generation of nanofibers from bottle-grade PET. Rajabinejad *et al.* generated fibers using the solvent-free process of melt-electrospinning [4]. Strain* et al.* dissolved the fibers in trifluoroacetic acid and dichloromethane and tested the fibers in an air filtration application [2].

Nanofibers, due to their very fine dimensions and high surface-area-to-volume ratio, are ideal candidates for a variety of high value applications including composites, optical and chemical sensing, protective textiles, photovoltaic cells, tissue engineering and drug delivery, and filtration. The most common method to generate nanofibers is through a facile process called electrospinning. In this process, the material solution or melt is forced through a needle charged at high voltage. Electrostatic repulsion in the droplet causes it to stretch and form what is known as a Taylor cone. Once the surface tension is exceeded, a fiber jet is formed that undergoes extensive stretching which leads to nanometer to micrometer sized fibers collected on the target [5,6].

World water shortages and the lack of accessibility of clean water of about 1/6th of our population suggests that new approaches to water cleaning and reuse are needed [7,8,9]. Due to the aforementioned characteristics of high surface-area-to-volume, fine fiber diameters and hence small pore sizes, in addition to high flexibility and surface modification potential, nanofiber membranes represent ideal candidates for next generation microfiltration materials [10,11]. Electrospun fibers have shown significant improvement in air filtration efficiency [12]. In terms of liquid filtration, porous membranes made by conventional methods have intrinsic limitations such as low-flux and high-fouling due to the physical structure of the pores and size distribution [13]. Electrospun membranes may overcome some of these limitations due to the highly interconnected and asymmetric nature of the pores.

Nanofiber use in liquid filtration has only recently been explored, but has shown great promise as a replacement material for commercial membranes. Nanofiber membranes have been applied in desalination, separation of solvents or oils and water, removal of heavy metals from ground water, and forward osmosis to name a few applications [14,15,16]. Yoon* et al.* generated composite filters with polyacrylonitrile nanofibers as the mid-layer and achieved a flux rate an order of magnitude higher than commercial nanofiltration membranes [17]. Bui *et al.* used blends of polyacrylonitrile and cellulose acetate for engineered osmosis membranes [18]. De Vrieze *et al.* tested polyamide nanofiber membranes functionalized with silver nanoparticles and biocides and found that 4 to 6 log of pathogenic bacteria were able to be removed [19]. Shin *et al.* used recycled polystyrene nanofibers for water-in-oil emulsion separations [20]. Zhao *et al.* generated a high flux filtration membrane from polyvinylidene fluoride and chitosan with a high rejection efficiency of bovine serum albumin [10].

In this work, we explore the use of electrospun recycled bottle-grade PET in the removal of particles ranging from 30 to 2000 nm. To the best of our knowledge, we are the first to report testing recycled PET (rPET) nanofibers in a liquid filtration application. The fibers were functionalized with biocidal materials to reduce biofouling and tested against the gram negative bacteria *Escherichia coli* (*E. coli*) and the gram positive bacteria *Staphylococcus aureus* (*S. aureus*).

## 2. Materials and Methods

### 2.1. Materials

Hexafluoroisopropanol (HFIP) was purchased from Oakwood Chemicals, Estill, SC, USA. Tributylammonium chloride (TBAC), tert-butanol, potassium carbonate, bromohexane, iodomethane, Tween 20, *N*-3-dimethylaminopropyl-*N*-ethylcarbondiimide (EDC), *N*-hydroxy succinimide (NHS), 2-*N-*morpholino ethanesulfonic acid (MES), hexanes and fluorescent latex beads (L5155, L9904, L5530, L9654, L9529) were purchased from Sigma-Aldrich, St. Louis, MO, USA. Trypticase soy broth, trypticase soy agar, phosphate buffered saline (PBS), and dey engley broth were purchased from Fisher, Waltham, MA, USA. *E. coli* (8739) and *S. aureus* (6538) were obtained from ATCC, Manassas, VA, USA. Vantocil 100 (B-1018-04) was purchased from Lonza, Allendale, NJ, USA. Lupasol WF (25,000 g/mol) was purchased from BASF, Florham Park, NJ, USA.

### 2.2. Quaternary Ammonium Polymer Synthesis

First, 5.5 g of Lupasol WF (25,000 g/mol) was dissolved in 70 mL tert-butanol and heated to 80 °C. Then, 21 g of potassium carbonate was added. Over a period of 2–3 min, 53 mL of bromohexane was added. The reaction was allowed to proceed for *ca.* 24 h, after which the potassium carbonate was removed via filtration. 15 mL of iodomethane was added and stirred at 60 °C in a closed container for 24 h. The polymer was then precipitated and washed with hexanes. Nuclear magnetic resonance (NMR) analysis was recorded on Bruker Avance 600 MHz NMR. CDCl_3_ was used as the solvent, referenced to residual solvent peak (7.26 ppm ^1^H, 77.15 ^13^C). ^1^H acquired with 32 scans, d1 = 2 s. ^1^H NMR of *N*-hexyl, *N*-methyl PEI in CDCl_3_ (d, ppm): 0.89 ppm (2H), 1.32 (3.7H), 1.81 (1H), 3.45 to 4.54 (5.2H). Peaks assigned as terminal methyl from hexyl chain (0.9), interior CH_2_ groups (1.3), b-CH_2_ groups (1.8), a-CH_2_ groups and methyl ammoniums, backbone peaks (3.4 to 4.5) [21].

### 2.3. Fiber Formation

For the recycled PET fibers, plastic water bottles were obtained from the recycle bin. After removal of the label, and the bottle was cut into strips with scissors and rinsed with water and ethanol and dried. The PET strips were then fed into a cross-cutting paper shredder. Then, 5–10 wt % solutions were prepared by dissolving the PET shreds in HFIP and stirring at room temperature overnight. TBAC (relative to the polymer weight) was added to the 5 wt % solution to reduce fiber bead formation.

All fibers were electrospun using a custom-built set-up consisting of a syringe pump (Aladdin AL-1000, World Precision Instruments, Sarasota, FL, USA) and a 4 inch aluminum collection plate. A 10-mL syringe was filled with polymer solution and fed through a 21-gauge stainless steel needle at a 1 mL/h flow rate with an applied potential of +17.5 kV at the needle. The gap between the needle and collector was fixed at 7 inches, and the collector was set to an applied potential of −3 kV. Fibers were vacuum dried overnight at RT before characterization.

### 2.4. Characterization of Nanofibers

The morphology of the fibers was examined using a field emission scanning electron microscope (SEM, Hitachi, Tarrytown, NY, USA) in the secondary-electron mode, using a mixture of upper and lower detectors. An accelerating voltage of 2 kV was maintained in order to prevent surface damage to the substrate. Before observation, the samples were vacuum dried and sputter coated with gold palladium. Several areas were imaged in order to examine the uniformity of the fiber diameters and pores. Pore size was determined by measuring the distance between the edge of one fiber to the edge of the next consecutive fiber (the distance between fibers). Fiber diameters and pores (*n* = 40) were measured using image analysis software (Image J v 1.34, National Institutes of Health, Bethesda, MD, USA).

The distribution of the fluorescent latex beads was probed using confocal laser scanning microscopy (CLSM) on a Zeiss LSM 700 (Zeiss, Thornwood, NY, USA). The beads were imaged with the 10× and 20× objectives using the 488 nm and 533 nm lasers. Fibers were imaged using phase contrast and overlaid with the fluorescent images.

Surface compositional analysis was performed using a Phi Versaprobe X-ray photoelectron spectroscopy (XPS, Physical Electronics, Chanhassen, MN, USA) system equipped with a hemispherical analyzer. Sampling areas of 100 µm^2^ were irradiated with a 140 W monochromatic Al Kα (1486.7 eV) beam and take-off angle of 90°. The XPS chamber pressure was maintained between 10^−9^ and 10^−10^ Torr. Elemental high resolution scans were conducted with a 20 eV pass energy for the C 1*s*, O 1*s* and N 1*s* core levels. A value of 284.6 eV for the hydrocarbon C 1*s* core level was used as the calibration energy for the binding energy scale. Data was processed using Casa XPS software (Casa Software Ltd., Teignmouth, UK). All reported atomic percentages are the average of *n* = 3 measurements.

### 2.5. Fiber Modification

The PET fibers were modified with a biguanide (Vantosil, Lonza, Allendale, NJ, USA) and a quaternary ammonium compound (Lupasol, BASF, Florham Park, NJ, USA) in order to reduce biofouling. For the covalent attachment of the antimicrobial molecules, the vacuum dried fibers were cut to 4 cm^2^ and plasma treated in air using an inductively coupled radio frequency plasma cleaner (Harrick PDC-32-G, Harrick Plasma, Ithaca, NY, USA) for 5 min at a power of 18 W to introduce carboxylic functionalities to the surface of the fibers. Plasma treated fibers were then immersed in a MES buffer containing 5 mg/mL EDC and 5 mg/mL NHS for 1 h at RT. Fiber mats were then rinsed with MES buffer and incubated in a 1 mg/mL solution of either Vantosil or Lupasol in PBS overnight at 4 °C. The solution was removed and the mats were washed in a 0.05% Tween 20 solution in PBS with gentle shaking for 30 min to remove physically absorbed material. For the physical adsorption of the antimicrobial material onto the fibers, the vacuum dried fibers were cut to 4 cm^2^ and immersed in a 1 mg/mL solution of either Vantosil or Lupasol in PBS overnight at 4 °C. All mats were then washed thoroughly with PBS and sterilized via ultraviolet light immediately before use, or rinsed with deionized water and dried for characterization.

### 2.6. Filtration Test

Mats of 5–10 wt % PET-R (18.2 ± 8.4 µm thick, 25.1 ± 11.6 g/m^2^) were cut to fit into a 1 inch diameter Buchner funnel. Three grams of a 200 ppm aqueous solution of latex fluorescent beads ranging from 30 to 2000 nm was passed through the filter using vacuum filtration using a pressure of 7.3 psi. The pressure was constant for all tests. The flux was calculated to be 230 ± 60 L·m^−2^·h^−1^·psi^−1^. The filtrate was analyzed using a fluorimeter. The percentage of beads removed or filtration efficiency was calculated by subtracting the fluorescence of the filtrate/ fluorescence of 200 ppm solution from 1. Mats were rinsed well and analyzed with CLSM or dried and sputter coated for SEM. All reported filtration efficiencies are the average a minimum of *n* = 3 trials. 

### 2.7. Antimicrobial Test

The antimicrobial properties of the rPET fibers were tested according to a modified AATCC-100 Test Method. The detailed protocol can be found in Sun *et al.* [22]. In brief, the fiber mats were cut to 4 cm^2^. One hundred microliters of 10^7^ colony forming units (CFU)/mL of bacteria were placed onto the surface of one mat and another mat was placed on top with active side touching the bacteria. After either 0 or 24 h, the mats were immersed in a neutralizing solution of Dey Engley broth, shaken for 5 min, serially diluted and plated. Percent reduction was calculated according to the AATCC-100 test method. All reported antimicrobial efficiencies are the average a minimum of *n* = 3 replicates.

## 3. Results and Discussion

**Figure 1:**
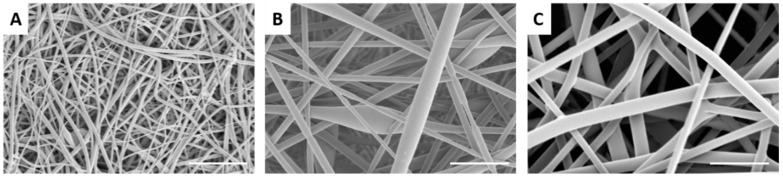
Scanning electron micrographs of electrospun recycled polyethlyene terephthalate (PET) nanofibers formed at different concentrations: (**A**) 5 wt %; (**B**) 7.5 wt %; and (**C**) 10 wt %. Scale bar denotes 5 µm.

Nanofibers were successfully prepared from recycled PET (rPET) solutions ranging from 5 to 10 wt %, as shown in Figure 1. Fiber diameters ranged from 105.5 ± 49 nm to 1039.5 ± 326 nm, with corresponding pore sizes of 244 ± 110 nm to 1427 ± 730 nm (5 to 10 wt %). TBAC salt was added to the 5 wt % solution to reduce the formation of beads [23]. Some beads were observed in the fibers formed from the 7.5 wt % solution, while beading was absent in the fibers formed from the 10 wt % solution. The fibers formed from the 10 wt % solution appear more web-like with many branching points evident, likely due to the high viscosity (345 cP). In previous work, we tested the mechanical properties of both the recycled and commercial off the shelf (COTS) PET with a fiber diameter of *ca.* 100 nm. We found that the recycled PET had higher elastic modulus and tensile strength compared to the COTS PET. Since fiber diameter and tensile strength are likely the dominant factors controlling filtration efficacy, the recycled PET nanofiber membranes are expected to have a similar if not better performance compared to COTS PET nanofibers membranes. However, testing COTS PET nanofiber membranes was outside the scope of this work.

**Figure 2:**
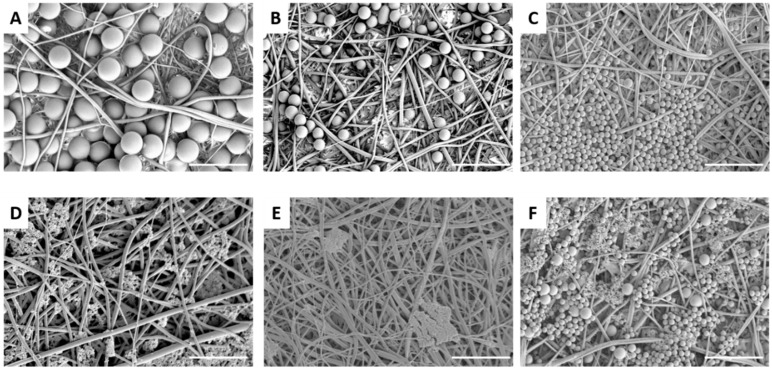
Scanning electron micrographs of electrospun 5 wt % recycled PET nanofibers after filtration with latex beads: (**A**) 2 µm beads; (**B**) 1 µm beads; (**C**) 500 nm beads; (**D**) 100 nm beads; (**E**) 30 nm beads; and (**F**) 30–2000 nm beads. Scale bar denotes 5 µm.

**Figure 3:**
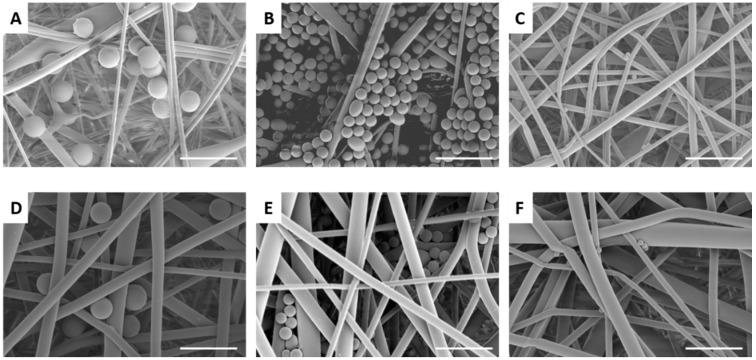
Scanning electron micrographs of electrospun 7.5 and 10 wt % recycled PET nanofibers after filtration with latex beads: (**A**) 2 µm beads; (**B**) 1 µm beads; (**C**) 500 nm beads; (**D**) 2 µm beads; (**E**) 1 µm beads; and (**F**) 500 nm beads. Scale bar denotes 5 µm.

**Figure 4:**
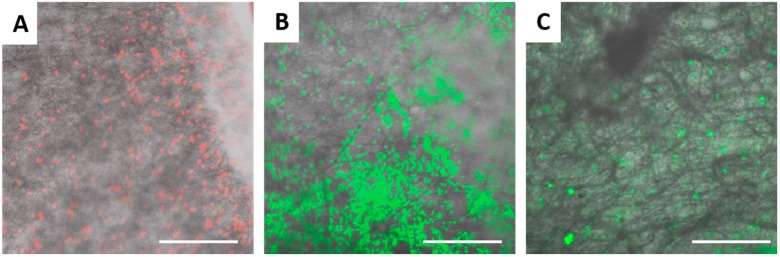
Confocal laser scanning images of electrospun 5 wt % recycled PET nanofibers after filtration with latex beads: (**A**) 2 µm beads (scale bar 80 µm); (**B**) 1 µm beads (scale bar 80 µm); and (**C**) 30 nm beads (scale bar 40 µm).

The rPET fiber mats were tested for particulate filtration efficiency using fluorescent latex beads of varied sizes. The results are displayed in Table 1. As expected the fibers with the smallest diameters and pore sizes (5 wt % rPET) captured the highest percentage of beads. The capture efficiency dropped significantly for the 100 nm and smaller beads, likely due to the larger average pore size of the fiber mats compared to the bead diameters. For the fibers formed from the 7.5 and 10 wt % solutions, filtration efficiency dropped significantly for the 500 nm and 1 µm beads, respectively. Figure 2 and Figure 3 display SEM images of the fiber mats post-filtration. For the fibers formed form the 5 wt % solution, the 1 and 2 µm beads are significantly larger than the pore sizes and remain primarily on the surface of the mat (Figure 2A,B). In contrast, the 500 nm beads appear to be captured by several layers of fibers (Figure 2C). The 100 nm beads are highly clumped and span the pores of the fibers, which accounts for the small amount of capture observed (Figure 2D). There is little evidence of the 30 nm beads on the fiber surface except for a few large clumps of tightly packed beads (Figure 2E). Figure 2F shows a mat in which a mixed solution of all 5 bead sizes was filtered. In Figure 3, the 1 and 2 µm beads appear smaller than the pore sizes for the larger rPET fibers (7.5% and 10% rPET), but the beads are trapped fairly efficiently due to the anisotropy of the fiber layers and non-overlapping pores. The 500 nm beads are generally not well captured since their diameter is significantly smaller than the average pore size of the mats (1030 ± 616 nm and 1427 ± 730 nn, 7.5 and 10 wt %). The reported *ca.* 30% capture rate is likely due to large clumps of beads. Confocal laser scanning microscopy was applied as an alternative method to study bead dispersion within the fiber mat (Figure 4). Again, a high level of capture of 2 and 1 µm beads is observed for the fibers formed from the 5 wt % rPET solution, whereas only clumps of beads are captured for the 30 nm beads. The effect of repeat use of the mats is shown in Table 2 for the 5% rPET fibers. The top half of the chart shows three filtrations done in a row without rinsing and the bottom with a water rinse in between. There is not a significant difference between the methods (*n* = 3) or a significant drop on capture efficiency with three filtrations. Thus, the mats are not clogging with several uses and do not require rinsing between filtrations, thereby increasing their ease of use. 

The flux was calculated to be 230 ± 60 L·m^−2^·h^−1^·psi^−1^, which is on the high end of the range compared to other reported fluxes from nanofibers membranes. This may be due to the thinness of the mat tested and lack of supporting substrate. Wang* et al.* reported a flux of 3.3 L·m^−2^·h^−1^·psi^−1^ for poly(vinyl alcohol) fibers in the separation of soybean oil/water emulsions [13]. Yoon found similar results for the filtration of oily wastewater with polyacrylonitrile fibers in a multilayer filter (flux of *ca.* 1 L·m^−2^·h^−1^·psi^−1^) [17]. Bjorge *et al.* reported a flux for clean water closer to the range we observed for a variety of membranes (140–400 L·m^−2^·h^−1^·psi^−1^) [24].

**Figure 5:**
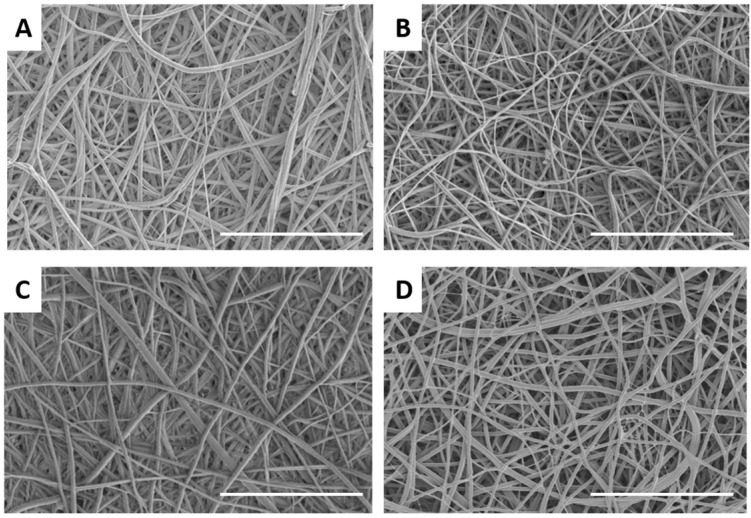
Scanning electron micrographs of electrospun 5 wt % recycled PET nanofibers after attachment of antimicrobial agents: (**A**) physically adsorbed Lupasol; (**B**) physically adsorbed Vantocil; (**C**) covalently attached Lupasol; and (**D**) covalently attached Vantocil. Scale bar denotes 10 µm.

**Figure 6:**
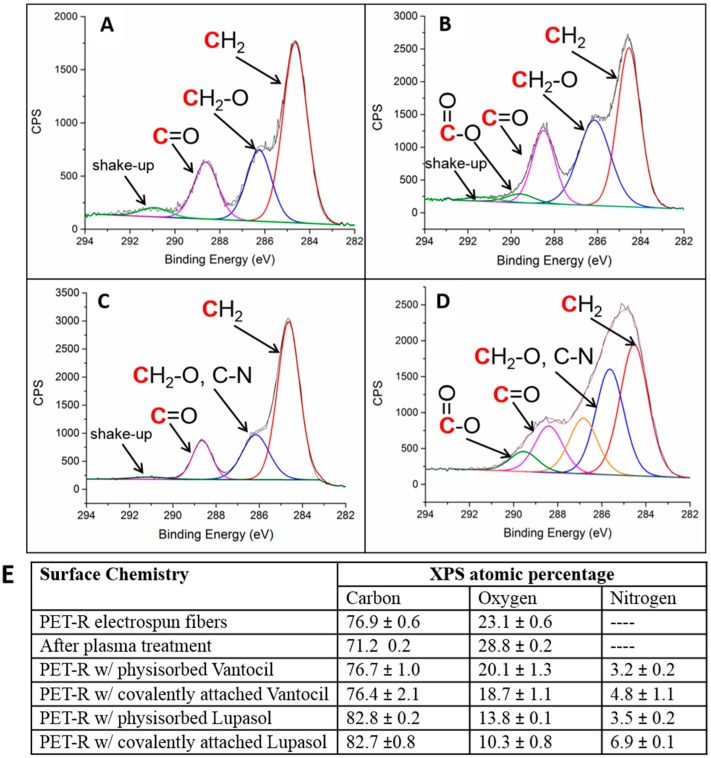
High-resolution X-ray photoelectron C 1*s* spectra of recycled PET fibers: (**a**) unmodified recycled polyethlyene terephthalate (rPET) fibers; (**B**) plasma-treated rPET fibers; (**C**) rPET fiber with physically adsorbed Vantocil; (**D**) plasma-treated rPET fibers with covalently attached Vantocil; and (**E**) quantification table.

To reduce biofouling and improve the lifetime of the filter mats, the surface of the fibers was coated with antimicrobial agents. Both a biguanide of *ca.* 3000 g/mol and quaternary ammonium polymer of *ca.* 25,000 g/mol were explored, which were either physically adsorbed or covalently attached to the surface. Both are classes of cationic materials that disrupt the cell membrane, leading to leakage of inner material and eventually cell death [25]. The Lupasol quaternary ammonium polymer was expected to have enhanced antimicrobial activity compared to the biguanide due to its higher molecular weight and hence larger number of charged sites. In addition, the Lupasol coating has already been approved by the Food and Drug Administration for both dry and wet food packaging, and thus approval for use in water filtration is likely [26]. The coating of both materials was uniform and thin, and did not significantly change the fiber diameter or morphology of the fibers as evidenced by Figure 5 and statistical analysis of the fiber diameter measurements. X-ray photoelectron spectroscopy was utilized to probe the surface chemistry of the fiber mats. Figure 6 displays high-resolution C 1*s* spectra of unmodified rPET fibers, plasma treated rPET fibers, rPET fibers with physically adsorbed Vantocil biguanide, and plasma-treated fibers with covalently attached Vantocil biguanide. The rPET C 1*s* spectrum has three main components in addition to an aromatic shake-up peak—the methylene, the ether/hydroxyl, and the ketone (Figure 6A). The plasma treated rPET has an additional component for the carboxylic acid from the plasma oxidation (Figure 6B). The biguanide C-N chemical shift falls in the range of the hydroxyl shift and thus no additional components are seen in Figure 6C,D. The elemental atomic percentages for both the attachment of the Vantocil biguanide and the Lupasol quaternary ammonium salt are displayed in Figure 6E. After plasma oxidation, the atomic percentage of oxygen increased from *ca.* 23% to 29%. Attachment of the antimicrobial agent led to the presence of nitrogen on the fiber surface, with the covalently attached antimicrobial agents exhibiting higher atomic percent nitrogen compared to their physically adsorbed counterparts.

The fiber mats were tested against gram negative *E. coli* and gram positive *S. aureus* using a modified AATCC-100 test as described in the Methods section [21]. The results are shown in Table 3. The rPET fibers without any surface treatment did not kill the bacteria. The quaternary ammonium salt Lupasol only killed the gram negative bacteria, while the biguanide Vantocil proved effective against both gram positive and gram negative bacteria. The covalently attached form was found to be the most effective for both the Lupasol and the Vantocil. This result was expected since the atomic percent nitrogen and thus antimicrobial agent on the fiber surface was higher for the covalently attached agents. The covalently attached form has the added benefit of not leaching from the surface over time, providing a longer lasting coating.

## 4. Conclusions

Nanofibers were fabricated from recycled PET and tested as liquid filtration membranes in order to assay whether more research using these membranes for this application was warranted. The pore size was found to scale with fiber diameter and hence the smallest fibers showed the best filtration capabilities. Although fiber diameters as low as *ca.* 100 nm were achieved, the mats were not suitable for ultrafiltration applications since particles under 500 nm were not effectively removed. However, the mats could be used in microfiltration applications such as a prefilter in a wastewater treatment system. Likely, a supporting layer will be needed due to the relatively low tensile strength of the nanofibers (*ca.* 5 MPa) unless used in very low pressure filtration applications [23]. Biofouling, a common problem with liquid filtration membranes, was markedly reduced with the surface modification of the fibers with cationic biocides. In particular, the covalent attachment of the Vantocil biguanide led to a 7 log reduction in both gram positive and gram negative bacteria. Thus, the nanofiber filters could potentially serve as a value-added application for recycled PET and a next-generation filter material.

## Figures and Tables

**Table 1:** Filtration efficiency of recycled PET nanofibers as determined by fluorescence spectroscopy.

wt % rPET	Percent Captured				
	2 µm Beads	1 µm Beads	0.5 µm Beads	0.1 µm Beads	0.03 µm Beads
5	99.7 ± 0.07	99.7 ± 0.01	99.3 ± 0.9	21.1 ± 6.2	1.64 ± 0.7
7.5	99.4 ± 0.3	99.3 ± 0.1	70.6 ± 4.3	-	-
10	89.9 ± 4.1	49.3 ± 19.2	27.8 ± 7.4	-	-

**Table 2:** Multiple filtration efficiency of recycled 5 wt % PET nanofibers as determined by fluorescence spectroscopy.

Rinse #	Percent Captured			
	2 µm Beads	1 µm Beads	0.5 µm Beads	0.1 µm Beads
1	95.2 ± 5.2	99.1 ± 0.4	97 ± 3.6	13.6 ± 5.6
2	96.7 ± 2.7	99 ± 0.3	98.6 ± 1.1	6.4 ± 2.6
3	96 ± 5.6	99.2 ± 0.2	99.1 ± 0.2	8.9 ± 2.7
1 *	99.2 ± 0.4	95.6 ± 1.4	98.9 ± 0.2	-
2 *	96.7 ± 4.3	91.8 ± 8.5	99.2 ± 0.4	-
3 *	97.6 ± 2.9	83.4 ± 14.2	99 ± 0.5	-

* Mats were rinsed with water between filtrations.

**Table 3:** Antimicrobial efficacy of attached antimicrobial agents on recycled PET nanofibers.

Surface Treatment	Percent Reduction	
PET nanofibers	*E. coli*	*S. aureus*
None (control)	NR	NR
Lupasol (covalent)	99.9	NR
Lupasol (physically adsorbed)	26.7	NR
Vantosil (covalent)	100	100
Vantosil (physically adsorbed)	100	97.7

NR denotes no reduction.
